# Variation of leaf shape with tree size: a case study using *Camptotheca acuminata* Decne

**DOI:** 10.3389/fpls.2024.1468483

**Published:** 2024-11-20

**Authors:** Ke He, David A. Ratkowsky, Pengjiazi Fu, Weihao Yao, Meng Lian, Long Chen, Peijian Shi

**Affiliations:** ^1^ School of Architecture, Huaqiao University, Xiamen, China; ^2^ Bamboo Research Institute, Nanjing Forestry University, Nanjing, China; ^3^ Tasmanian Institute of Agriculture, University of Tasmania, Hobart, TAS, Australia; ^4^ Design and Research Institute, Shenzhen University, Shenzhen, China

**Keywords:** centroid ratio, leaf area, leaf roundness index, power-law equation, proportional relationship

## Abstract

The Montgomery equation (ME) assumes that leaf area (*A*) is proportional to the product of leaf length (*L*) and width (*W*). Leaf shape is found to determine the ME’s proportionality coefficient, i.e., the Montgomery parameter (MP). However, prior work seldom reported the influence of tree size (reflected by the diameter at breast height, DBH) on leaf shape and size. In the present study, we sampled 840 leaves from six trees of *Camptotheca acuminata*, with 140 leaves for each tree. Three leaf-shape indices were measured for each leaf, viz. the width to length ratio (*W*/*L*), a leaf roundness index which indicates the extent to which the leaf shape approaches a circular leaf, and the centroid ratio, defined as *l*/*L*, where *l* is the distance from the leaf base to the point on the leaf length axis where the leaf width is a maximum. For each tree, the ME was investigated in two ways, one being that *A* was assumed to be proportional to the product of *L* and *W*, and the second being a power-law equation which assumed an allometric relationship between *A* and *LW*, i.e., *A* ∝ (*LW*)^α^, where α is a constant to be estimated. The centroid ratio slightly decreased with increasing DBH, indicating that larger trees tend to have more ovate leaves than elliptical leaves. However, DBH did not significantly affect the ratio *W*/*L* nor the leaf roundness index. The estimated MP for the pooled data was 0.6466, and it was not statistically affected by DBH. The numerical value of α was found to approximate unity. The percent error between ME and the power-law equation was smaller than 5%, which means that there is no need to use the power-law equation to describe the relationship between *A* and *LW*. ME is valid for the calculation of *A* at the individual tree level and for the pooled data of all trees. The present study indicates that the influence of DBH on MP can be neglected when calculating *A*, and any easily accessible trees can be selected to examine the *A* versus *LW* isometric relationship.

## 1 Introduction

**Figure 1:**
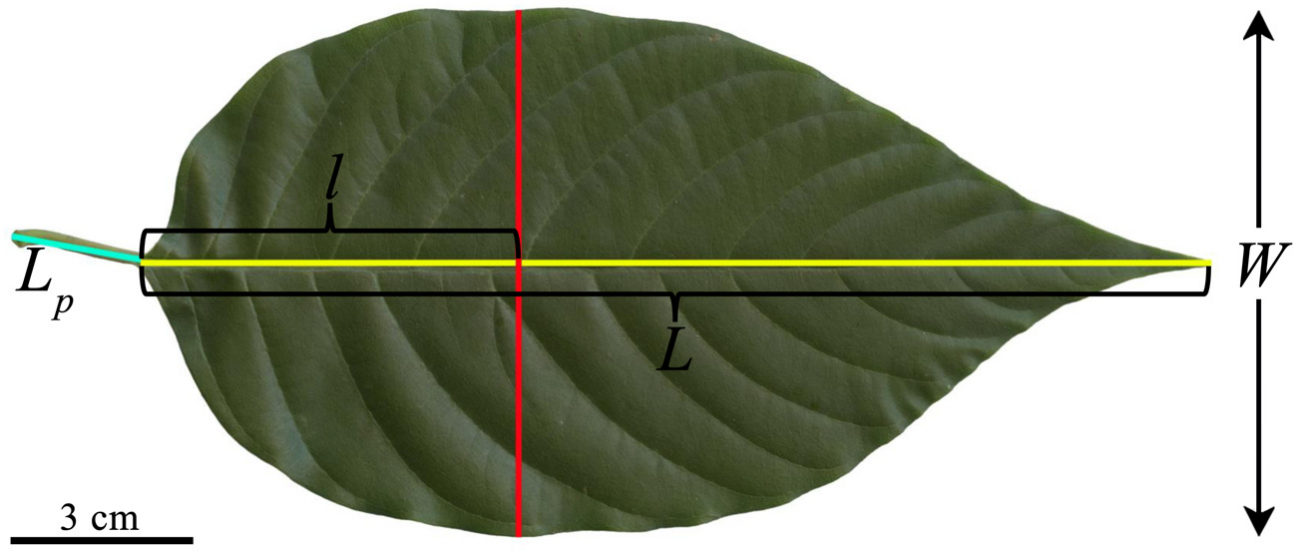
Illustration for the definitions of some one-dimensional leaf measures of *C. acuminata*, including leaf petiole length (*L_p_*), leaf (lamina) length (*L*), leaf (lamina) width (*W*), and the distance from leaf base to the point on the leaf length axis associated with *W*, denoted as *l*. Here, the leaf image was transferred to the black and white.bmp image, and the Matlab (version ≥ 2009a; MathWorks, Natick, MA, USA) procedure developed by Su et al. (2019) was then used to obtain the planar coordinates of the leaf boundary. Leaf area (*A*), perimeter (*P*), *L*, *W*, and *l* were calculated using the “bilat” function of the “biogeom” package (version 1.3.5) based on the statistical software R (version 4.2.0). Then the three leaf-shape indices were obtained for the leaf. The leaf centroid ratio is defined as *l/L*; the ratio of leaf width to length equals *W/L*; and the leaf roundness index is defined as 4π*A*/*P* ^2^.

Leaf shape and size can determine the efficiency of light interception and photosynthetic rate of plants (Niklas, 1989, Niklas, 1999; Smith et al., 1997; Nicotra et al., 2008, Nicotra et al., 2011). If the growth season of plants is short, e.g., the plants growing under cold climates, the photosynthesis is stronger than those growing in subtropical and tropical areas (Royer and Wilf, 2006). Leaf shape should be designed to help to dissipate heat, and plants growing in cold climates do not tend to have entire leaf edge as more have leaf dissection, lobes, margin serration and margin toothiness (Royer and Wilf, 2006). Leaf area and the ratio of leaf mass to leaf area (i.e., leaf mass per unit area) are directly linked to light interception and photosynthetic rate of plants. Larger leaves can intercept more light, and leaf mass per unit area means higher chlorophyll content and stronger photosynthetic rate (Poorter et al., 2009; Miao, 2024). Tree growth and longevity, which can be reflected by tree size (e.g., height, stem diameter, and crown size), are key features in understanding fundamental issues of plant biology, environmental sciences and forest management plans (Munné-Bosch, 2018). Due to the inter- or intra-specific competition for light, trees tend to fast grow, given that taller trees shade shorter trees but not vice versa (Falster and Westoby, 2003). However, the tradeoff between the increased path length that water travels and the increased gravitational resistance of taller trees is necessitated to compensate losses of foliage due to the hydraulic limitation (Midgley, 2003). Root pressure and vessel size largely determine plant height and leaf size (Wang et al., 2011; Cao et al., 2012). Leaf shape and size might vary with tree size increasing as a response to hydraulic limitation. Leaf venation pattens and leaf shape influence each other, and leaf venation patterns can determine the efficiency of water use (Runions et al., 2017; Sack and Scoffoni, 2013). It is apparent that the hydraulic limitation for tree height is likely to have an influence on leaf shape and size via adjusting the water use in leaves. However, little is known about the influence of tree size on leaf shape and size. Prior work shows that leaf shape and size largely vary at inter- and intra-specific levels (Wright et al., 2017; Baird et al., 2021; Schrader et al., 2021). Even for the same plant species, leaf size is also likely to exhibit a large variation within a plant, e.g., the sun leaves tend to be small and thick, and the shade leaves tend to be large and thin (Dörken and Lepetit, 2018). Temperature, rainfall and solar radiation have been found to be the key drivers of leaf size around the world, and the drivers account for both the giant leaves of tropical plants and the tiny ones of desert dwellers (Wright et al., 2017). For different species or different geographical populations of the same species, the variation in leaf size mainly reflects the influence of these environmental conditions on plant ecology and evolution. The intraspecific variation in leaf size for the same species growing in the same site subjected to the same growing conditions can better reflect the survival strategies of plants, especially different age groups of the same plant species, e.g., saplings and adults, adapting to environment and competition (Liu et al., 2020; Huang et al., 2021). Young trees invest more biomass in increasing leaf area than adults (Liu et al., 2020). Relative to leaf size, leaf shape appears to be relatively stable at the same growth stage of leaves. However, the quantification of leaf shape is somewhat challenging given the large variation in leaf shape. There are some indices to quantify leaf shape, and the most-commonly used indices are probably the ratio of leaf width to length, leaf roundness index and leaf dissection index (Thomas and Bazzaz, 1996; Niinemets et al., 2004, Niinemets et al., 2005; Peppe et al., 2011; Shi et al., 2021a). In general, leaf bilateral symmetry can influence the numerical values of the above three leaf-shape indices to a large degree. Prior work shows that leaf bilateral symmetry tends to reflect the influence of light on the above-ground architecture of plants (Wang et al., 2018). Several indices were proposed to quantify the leaf bilateral symmetry including the ratio of the left side’s area to the right side’s area, and the standardized index for leaf bilateral asymmetry (Palmer and Strobeck, 1986; Shi et al., 2020; Mu et al., 2024), which are both regarded as measures for leaf shape. In the present study, we focus on ovate leaves that are found in almost all biomes (Hickey, 1973; Ash et al., 1999). This leaf shape might reflect the trade-off between the leaf radial growth around midrib and hydraulic constraints of vascular plants (Yang et al., 2022). Prior work defined a leaf-shape index for ovate leaves, denoted as the centroid ratio (Shi et al., 2021b). This index equals the ratio of the distance *l* (from the leaf base to a point on the leaf length axis associated with maximum leaf width) to leaf length *L* (see **Figure 1** for details). Ma et al. (2022) reported that with tree size increasing, the centroid ratio did not change linearly while the ratio of leaf width to length tended to become larger. However, no other studies have examined the influence of tree size on the centroid ratio of ovate leaves.

Leaf mass, leaf area, leaf length, leaf width, and leaf thickness can all represent leaf size, because these measures of leaf size are positively correlated (Lin et al., 2018). The use of leaf mass to quantify leaf size usually involves destructive sampling, which is impractical for field investigation. Leaf area is a better choice because it was demonstrated to be proportional to the product of leaf length and width, an early conclusion when calculating the leaf area of corn (Montgomery, 1911). We refer to it as the Montgomery equation (ME), and the proportionality coefficient of ME as the Montgomery parameter (MP). Prior studies found that the numerical value of MP usually ranged from 1/2 to π/4, i.e., varying between a triangular leaf and an elliptical leaf (Jani and Misra, 1966; Palaniswamy and Gomez, 1974; Robbins and Pharr, 1987; Verwijst and Wen, 1996; Salerno et al., 2005; Koyama et al., 2012; Shi et al., 2019; Shi et al., 2021a; Yu et al., 2020; Li et al., 2022a; Li et al., 2022b). Nevertheless, for many vines with concave leaves caused by lobes, and for hyper-elliptical leaves, MP can fall outside the range 1/2 to π/4 (Mu et al., 2024). However, there is a need to test whether plant size and age can influence leaf shape that can be gauged by the MP. Only a few studies have examined this topic (de Swart et al., 2004; Huang et al., 2021; Ma et al., 2022). Huang et al. (2021) found that culm age of moso bamboo (*Phyllostachys edulis*) had little influence on the MP of bamboo leaves. Ma et al. (2022) found that the diameter at breast height (DBH, representing tree size) of *Quercus pannosa*, an alpine oak, can influence the leaf shape, but not MP across different tree-size groups. However, considering that only a few studies have reported the influence of tree size on the leaf shape and MP, there is still a need to consider results from other case evidence.

Plant size can be reflected by many measures including plant height, diameter at breast height (DBH), ground diameter, crown size (usually denoted by a product of two one-dimensional measures, i.e., the length from south to north multiplied by the length from east to west of the crown). In traditional forest survey especially for those dense forests, it is time-consuming and inconvenient for accurately measuring tree height. Thus, DBH is usually used for representing tree size (Zhang et al., 2024). Such a representative is found to be reasonable because the DBH values for trees and taller bamboos are found to follow the three-parameter Weibull distribution (Cheng et al., 2015; Zhang et al., 2024).

In the present study, we selected six trees of *Camptotheca acuminata* Decne with different DBH values and tree height, and sampled 140 leaves from each tree to test whether DBH can significantly influence leaf shape and size, and the relationship between leaf area and the product of leaf length and width. The study objectives are associated with the test on whether the hydraulic limitation for height growth can influence leaf morphology and size. We have the following two points of consideration for choosing *C. acuminata* as the study material: (i) the trunk of the tree species is straight and high, which is applicable for reflecting the applicability of leaf functional traits to height competition; (ii) the tree species has typical ovate leaves, which is suitable for measuring the above leaf-shape indices especially leaf centroid ratio. If the influence of DBH on the proportional relationship between leaf area and the product of leaf length and width can be neglected, this means that the proportionality coefficient (i.e., MP) before the product of leaf length and width that is used to calculate leaf area exhibits a negligible variation across different tree sizes. Then any easily accessible trees can be selected to calculate leaf area directly using the ME regardless of the difference in tree size. This work is different from Chen et al. (2024) using these materials. Chen et al. (2024) examined the influence of the DBH values on the scaling relationship of leaf mass versus leaf area, which gauged the tradeoff between biomass investment to support costs and photosynthetic returns (Niklas et al., 2007).

## 2 Materials and methods

### 2.1 Tree and leaf sampling information

Six *C. acuminata* trees were selected from a larger set of individual trees growing in the Nanjing Forestry University Xinzhuang campus (118°49′17″ E, 32°04′42″ N) whose terrain is fairly level. We selected the six trees with different DBH values growing close to roads for conveniently sampling leaves. This species was selected for study because of the availability of trees differing in size but growing under nearly identical horticultural conditions. The diameter at breast height (DBH) values are 15.60, 19.09, 23.25, 40.12, 42.36, and 44.74 cm, and the corresponding tree height values are 11.50, 12.10, 13.70, 16.50, 18.10, and 18.50 m. Compared to tree height measured using DJI drone (Mavic Mini 1, DJI, Shenzhen, China), the DBH were more accurately measured by a ruler. We sampled 100 leaves from the lower canopy of each tree (i.e., the bottom third of each tree crown) without considering branch growth orientation (i.e., the leaves were randomly sampled from all around the tree crown’s lower layer) from July 18th to July 20th, 2023. To carry out more robust data analysis, we additionally sampled 40 leaves for each tree on 3 September, 2024. The same sampling protocols were performed. In spite of the fact that leaf area and the scaling relationship between leaf area and leaf mass can vary across different age groups, leaf shape and the isometric relationship between leaf area and the product of leaf length and width exhibited little variation (Liu et al., 2020; Jiao et al., 2022; Zheng et al., 2022; Guo et al., 2023). Thus, it is reasonable to combine the data in two samplings. The lower canopy is an easily accessible position for leaf sampling given the heights of the six trees. And there is no evidence to show that leaf shape in the bottom third of the tree crown is significantly different from that in the top third and in the middle third. The sampled leaves, wrapped in wet paper, were placed in resealable plastic bags (28 cm × 20 cm), and brought back to the laboratory within two hours. The raw data of the 600 leaves sampled in 2023 can be accessible in Chen et al. (2024), and the data of the 240 leaves sampled in 2024 are listed in online **Supplementary Table S1.**

### 2.2 Data acquisition

The petiole of each fresh leaf was removed using a sharp blade, and the remaining lamina was scanned to a .jpg image using a photo scanner (V550, Epson, Batam, Indonesia). The scanned .jpg images were transformed into black and white .bmp images using Adobe Photoshop CS6 (version 13.0; Adobe, San Jose, CA, USA). The Matlab (version ≥ 2009a; MathWorks, Natick, MA, USA) procedure developed by Su et al. (2019) was then used to obtain the planar coordinates of each leaf boundary. Leaf area (*A*), length (*L*), width (*W*), perimeter (*P*), and the distance from the leaf base to the point on the leaf length axis associated with *W*, denoted as *l* (see **Figure 1** for details), were calculated using the “bilat” function of the “biogeom” package (version 1.3.5; Shi et al., 2022) based on the statistical software R (version 4.2.0; R Core Team, 2022).

### 2.3 Leaf-shape indices

In the present study, three leaf-shape indices comprising the leaf centroid ratio (CR), the ratio of *W/L*, and the leaf roundness index (RI) were used to quantify leaf shape complexity. CR is defined as *l/L*, and RI is defined as 4π*A*/*P* ^2^, the latter ranging between 0 and 1. For an ovate leaf, a large CR means that the point on the leaf length axis associated with leaf maximum width (i.e., *W*) is close to the midpoint of *L*, indicating the leaf shape is more elliptical. When RI approximates unity, it indicates a rounder leaf. R (version 4.2.0; R Core Team, 2022) was used to calculate all leaf-shape indices.

### 2.4 Data analyses

The analysis of variance (ANOVA) with Tukey’s honestly significant difference (HSD) test with a 0.05 significance level (Hsu, 1996) was used to test the significance of the differences in each of the above leaf-shape indices between any two trees. Linear regression (see Equation 1) was carried out to test whether tree size (reflected by DBH) influenced leaf size (reflected by mean leaf area) and leaf shape (reflected by mean CR, mean ratio of *W/L*, and mean RI), where *x* is DBH, *y* is the mean of leaf area or leaf shape measures, *a* is the intercept, and *b* is the slope. We tested whether DBH has a significant influence on *y* by examining whether the slope is statistically significant.


(1) y=a+bx,

The Montgomery equation (ME; Montgomery, 1911; Shi et al., 2019; Schrader et al., 2021) was used to fit the relationship between *A* and the product of leaf length and width (*LW*) for each tree separately and for the pooled data of the six trees, i.e.,


(2) A=kLW,

where *k* is the Montgomery parameter (MP) to be estimated. To stabilize the variance, the log-transformation was used, so that ME takes the form:


(3) log(A)=c+log(LW),

where *c* is log(*k*). Ordinary least-squares was used to estimate the parameter *c*. The bootstrap percentile method (Efron and Tibshirani, 1993; Sandhu et al., 2011) was used to test the significance of the difference in the MP values between any two trees. 3000 bootstrap replicates of MP for each tree were generated, and the 95% confidence interval (CI) of the differences in the MP replicates between any two trees was calculated. If the CI doesn’t include zero, it indicates a difference at the 0.05 significance level, otherwise not. MP is also recommended as a leaf-shape index to quantify the deviation of leaf shape from an ellipse (Li et al., 2022b). However, there is no mathematical relationship between MP and other leaf-shape indexes including CR, *W/L* and RI. CR is used to quantify the deviation of the location on the leaf length axis associated with the maximum width deviating from the midpoint of the leaf length axis; *W/L* is used to measure the ratio of leaf two one-dimensional maximum measures in perpendicular orientation regardless of where the maximum leaf width is located, which is the focus of CR; RI is used to quantify the deviation of leaf shape from a circle (Niinemets et al., 2004, Niinemets et al., 2005; Peppe et al., 2011).

Given that leaf surface is strictly not flat, we tested whether a power-law equation was better than ME (Niklas et al., 2007; Shi et al., 2019; Yu et al., 2020):


(4) $$A=\beta {\left(LW\right)}^{\text{α}},$$

where *β* is the normalized constant, and α is the scaling exponent. As before, to stabilize the variance, the log-transformation was used, and Equation 4 takes the form as


(5) log(A)=γ+α log(LW),

where γ is log(β). Ordinary least-squares was used to estimate the intercept γ and slope α .

The root-mean-square error (RMSE) was used to reflect the goodness of fit for ME and the power-law equation (i.e., Equations 3, 5), and takes the form:


(6) $$\text{RMSE}=\sqrt{\frac{\sum_{i=1}^{n}{\left(y_{i}-{\hat{y}}_{i}\right)}^{2}}{n}},$$

To test whether to introduce an additional scaling exponent is worthwhile in Equation 5 compared with Equation 3, the percent error (PE) was calculated:


(7) $$\text{PE}=\frac{{\text{RMSE}}_{1}-{\text{RMSE}}_{2}}{{\text{RMSE}}_{1}}\times 100\%$$

where RMSE_1_ is the root-mean-square error of ME (i.e., Equation 3), and RMSE_2_ is the root-mean-square error of the power-law equation (i.e., Equation 5). RMSE_2_ ≤ RMSE_1_, and as a rule of thumb, we adopt PE ≥ 5% as indicating that the additional parameter α deserves to be introduced into the model (Yu et al., 2020), otherwise not.

## 3 Results

### 3.1 Effect of DBH on leaf size and shape

**Figure 2:**
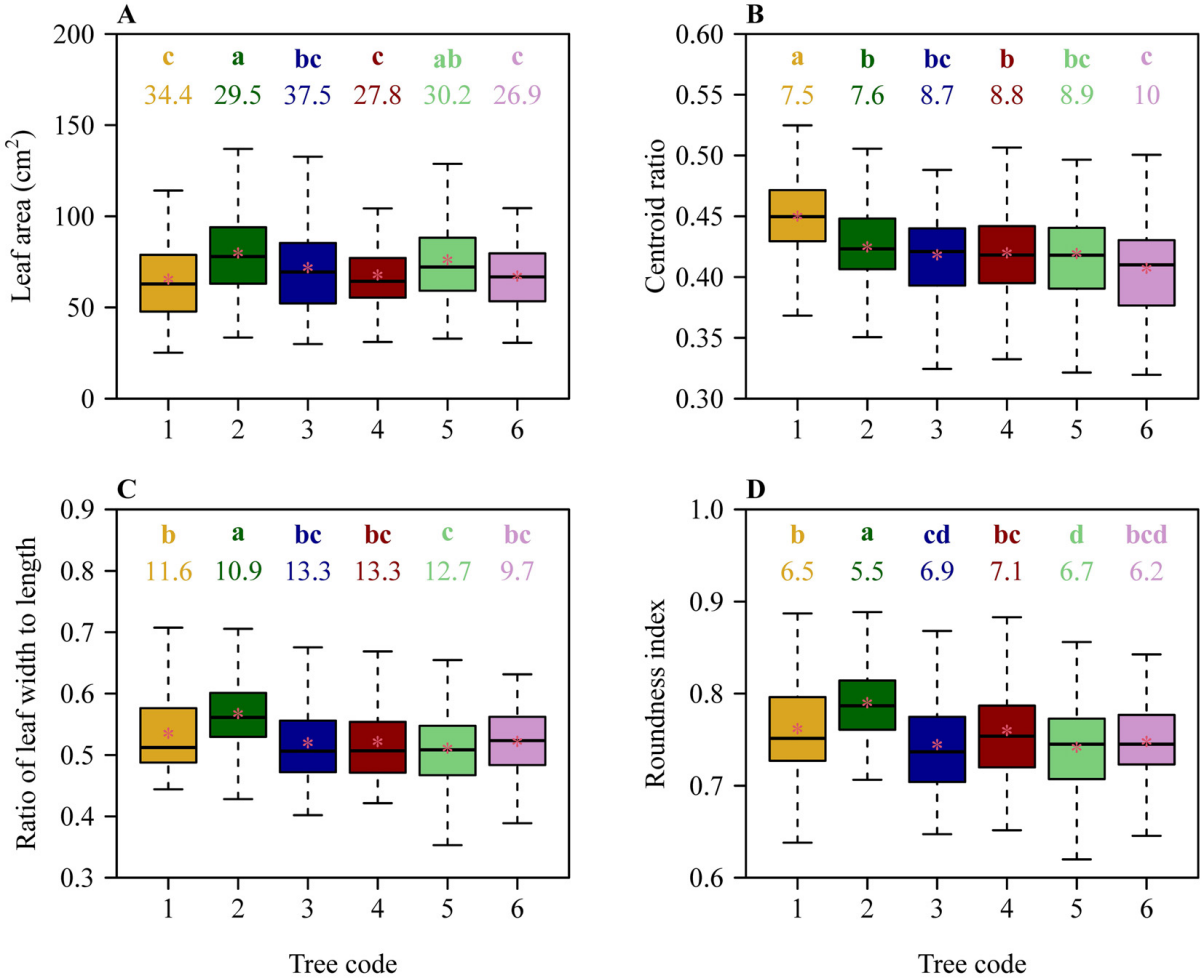
Comparisons of leaf size and shape among the six trees of *C. acuminata*. **(A)** Leaf area, **(B)** leaf centroid ratio, **(C)** the ratio of leaf width to length, and **(D)** leaf roundness index. The lowercase letters in each panel were used to indicate a significant difference of species based on Tukey’s HSD test (α = 0.05), and the numbers below the letters represent the coefficients of variation (%). The upper and lower ends of each box represent the 3/4 and 1/4 quantiles, respectively; the whiskers extend to the most extreme data point, which is no more than 1.5 times the interquartile range from the box; the horizontal bold lines in the boxes represent the medians; and the asterisks represent the means. Numbers from 1 to 6 on the *x*-axis label correspond to the diameter at breast height (DBH) values are 15.60, 19.09, 23.25, 40.12, 42.36, and 44.74 cm, respectively.

**Figure 3:**
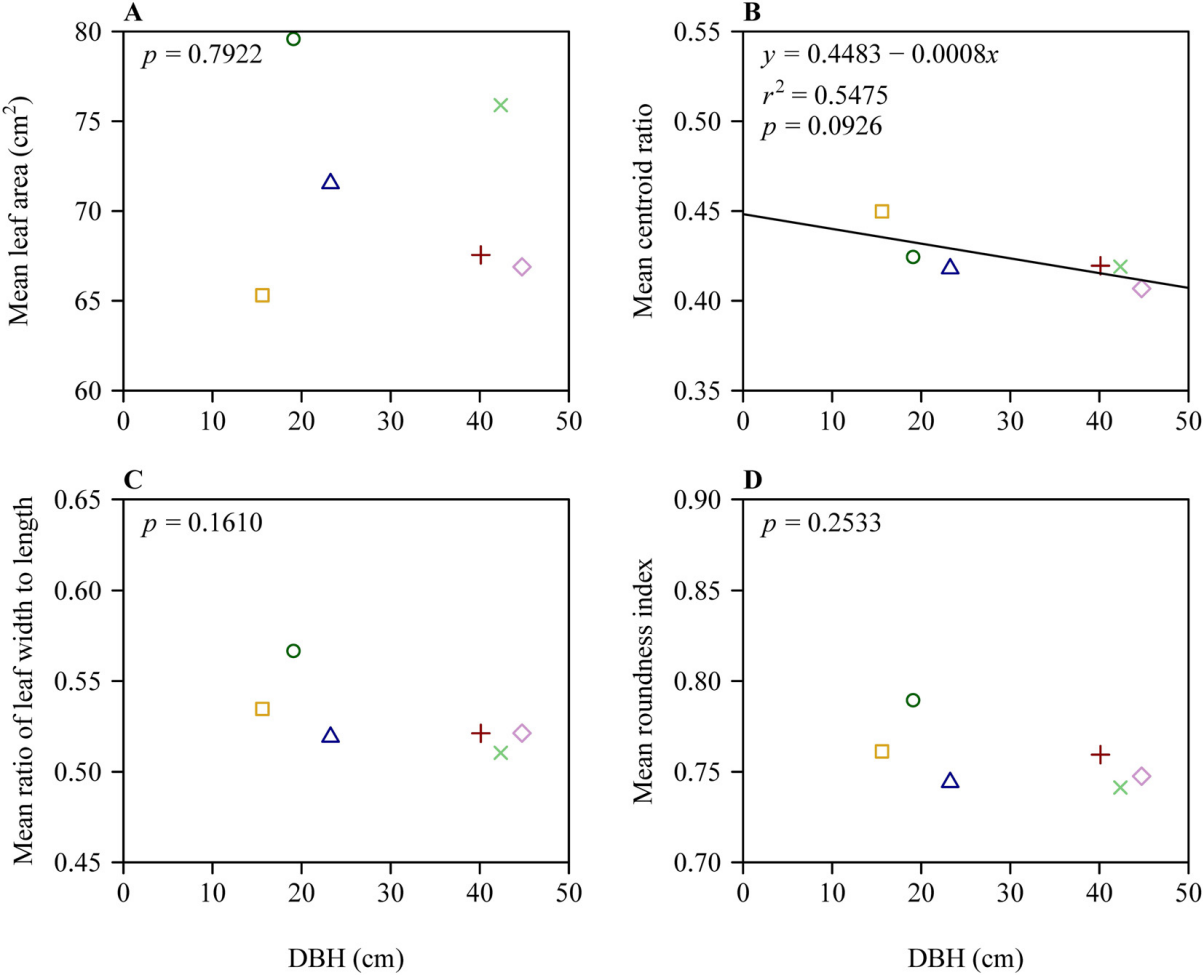
Results of fitting the linear equation of mean leaf size and leaf shape measures vs. the diameter at breast height (DBH). **(A)** Mean leaf area vs. DBH, **(B)** mean centroid ratio vs. DBH, **(C)** mean ratio of leaf width to length vs. DBH, and **(D)** mean roundness index vs. DBH. In each panel, different icons represent the means for leaf size or shape from the six trees; the straight line represents the regression line.

There were significant differences in leaf size and shape among the six trees based on Tukey’s HSD test (**Figure 2**). Despite this, results of the linear regression of leaf size and shape vs. DBH showed that the slopes of mean leaf area vs. DBH, mean *W/L* ratio vs. DBH, and mean RI vs. DBH were not significantly different from zero (in each of these 3 cases, *p* > 0.05). However, the slope of mean CR vs. DBH exhibited a weak significance (*r* ^2^ = 0.55; *p* = 0.0926 < 0.10) (**Figure 3**).

### 3.2 Effect of DBH on the relationship between leaf area and the product of leaf length and width

**Figure 4:**
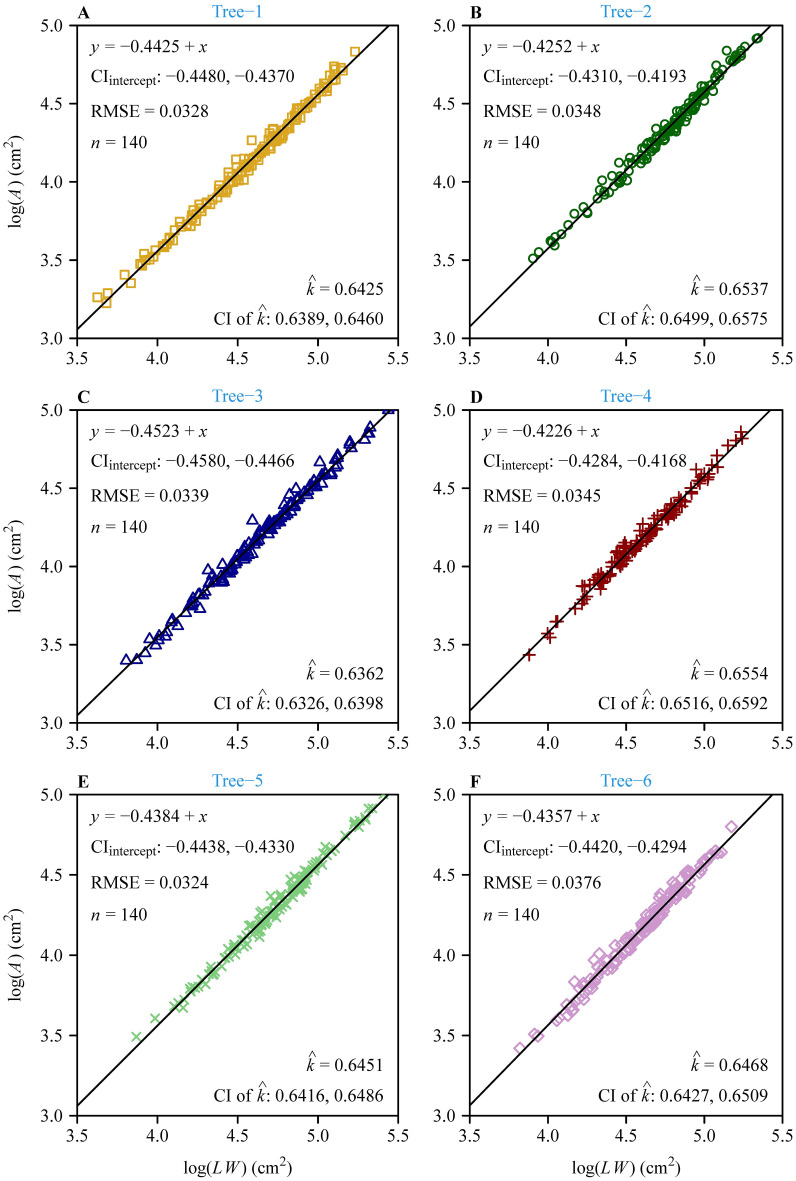
Results of fitting the Montgomery equation for each of the six trees. Here, panels **(A-F)** represent the six trees with different diameter at breast height (DBH) values. In each panel, *A*, *L*, and *W* represent the leaf area, length, and width, respectively; *y* represents log(*A*), and *x* represents log(*LW*); CI_intercept_ represents the 95% confidence interval of the intercept; RMSE represents root-mean-square error; *n* represents the number of data points; $\hat{k}$ represents the estimate of the Montgomery parameter; CI represents the 95% confidence interval of the Montgomery parameter based on 3000 bootstrap repetitions. Different icons represent the observations from different trees, and the straight line represents the regression line.

**Figure 5:**
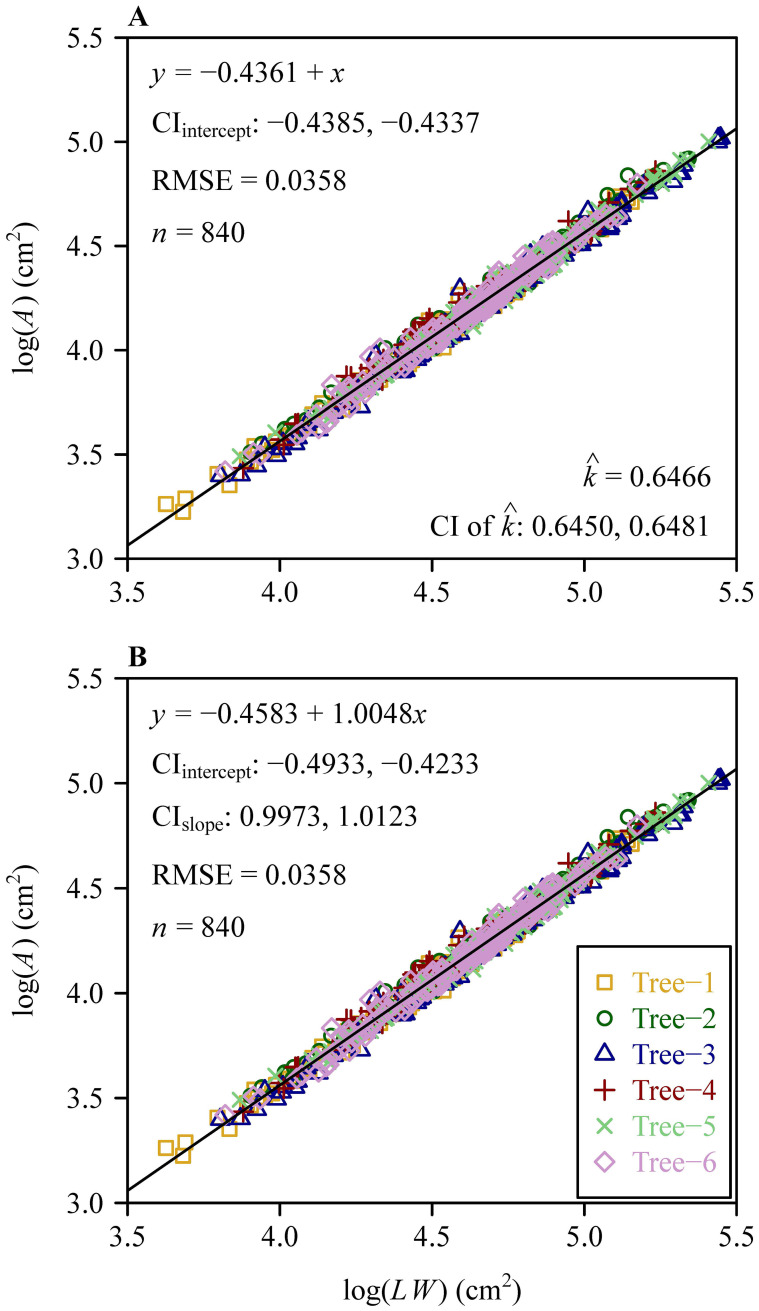
Results of fitting the Montgomery equation and the power-law equation for the pooled data of the six trees. Here, *A*, *L*, and *W* represent the leaf area, length, and width, respectively; *y* represents log **(A)**, and *x* represents log(*LW*); CI_intercept_ represents the 95% confidence interval of the intercept; RMSE represents root-mean-square error; *n* represents the number of data points; $\hat{k}$ represents the estimate of the Montgomery parameter; CI represents the 95% confidence interval of the Montgomery parameter based on 3000 bootstrap repetitions. Different icons represent the observations from different trees, and the straight line represents the regression line. In panel **(B)**, CI_slope_ represents the 95% confidence interval of the slope.

**Figure 6:**
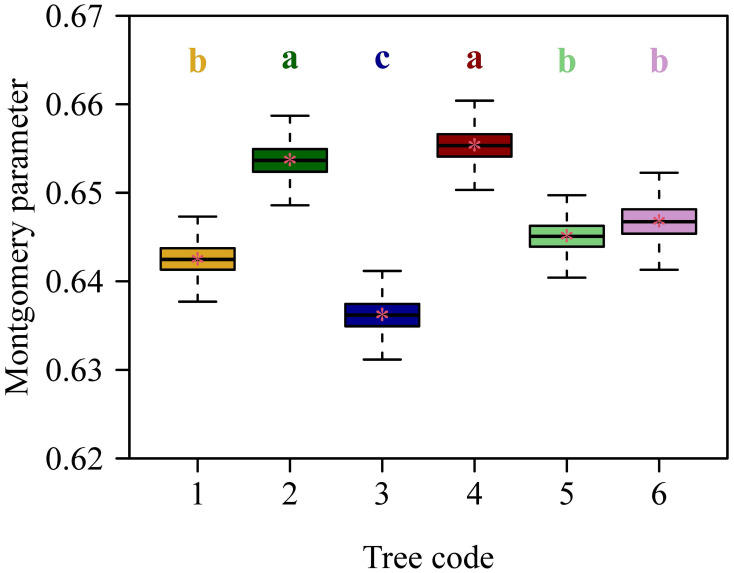
Estimates of the Montgomery parameter (MP) among the six trees. The upper and lower ends of each box represent the 3/4 and 1/4 quantiles, respectively; the whiskers extend to the most extreme data point, which is no more than 1.5 times the interquartile range from the box; the horizontal bold lines in the boxes represent the medians. The letters a, b, and c on the top of each upper whisker display the significance of differences between any two trees. The order of the letters in the alphabet denotes MP values sorted from the highest to the lowest.

The Montgomery equation (ME) was validated for each tree, and the RMSE values ranged from 0.0324 to 0.0376, all smaller than 0.05 (**Figure 4**). The RMSE value of ME for the pooled data was equal to 0.0358, which was also smaller than 0.05 (**Figure 5**). This demonstrated the validity of ME for describing the relationship between *A* and *LW* regardless of DBH. The MP values ranged from 0.6362 to 0.6554 for the six trees, and had negligible differences; the MP value for the pooled data was equal to 0.6466. The PE values between ME and the power-law equation for the six trees were all smaller than 5%, and the PE value for the pooled data was also smaller than 5%. Additionally, the point estimate and the lower bound of the 95% confidence interval of the *A* vs. *LW* scaling exponent (i.e., α in Equation 5) were approximately equal to unity, which implies an isometric relationship rather than an allometric relationship between *A* and *LW*. This indicates that there is a negligible need to introduce an additional scaling exponent in describing the relationship between *A* and *LW*. In addition, the estimated values of MP differed statistically significantly among the six trees based on Tukey’s HSD test (**Figure 6**), but the slope of the estimates of MP vs. DBHs was not statistically significant (*p* = 0.6014).

## 4 Discussion

In the present study, leaf size was found not to exhibit a linear change with increasing DBH, as well as two leaf-shape indices (i.e., the *W/L* ratio and leaf roundness index [RI]). Nevertheless, the centroid ratio (CR) decreased with increasing DBH. This means that the three leaf-shape indices have different responses to increased DBH. In this section, we mainly focus on the probable reasons for the results. In addition, we concern ourselves with which factor determines the numerical value of the Montgomery parameter (MP).

### 4.1 Why did the three leaf-shape indices respond differently to DBH?

In the present study, three leaf-shape indices, the CR, *W/L* ratio, and RI, were used. However, only CR exhibited a linear decrease with increasing DBH. These results are easy to account for, as the three leaf-shape indices measure different quantities. An ovate leaf usually has CR ranging between 0 and 0.5. If CR exceeds 0.5, the leaf shape tends to be obovate. In the 840 leaves, there were only 15 leaves with CR > 0.5. For the remaining 825 leaves, with CR approaching 0.5, the leaf resembles an ellipse or a superellipse (Li et al., 2022a). However, the *W/L* ratio cannot determine whether a leaf shape is more elliptical than another leaf, and is used only to determine whether a shape is narrow or broad. Leaf RI, which ranges between 0 and 1, is used only to reflect whether a shape tends to be circular. When RI = 1, the shape is a circle; when RI = 0, the shape is a line. For an ellipse with a small ratio of the minor-axis length to the major-axis length, RI is small. This means that RI is not used to determine whether a shape tends to be elliptical or not, just to determine whether a shape tends to be round or not (Shi et al., 2020). The *W/L* ratios of the six studied trees ranged from 0.35 to 0.85 (**Figure 2C**), so the ovate leaf shape is not a circle (whose *W/L* ratio should equal unity). Thus, RI has a limitation in quantifying ovate and elliptical leaf shapes. Ma et al. (2022) found that the *W/L* ratio but not CR tends to increase with increasing DBH for an alpine oak (*Quercus pannosa*) whose leaf shape tends to be elliptical rather than ovate. Thus, for elliptical or oblong leaves, it is the *W/L* ratio that reflects leaf size, whereas for a typical ovate leaf shape, as for *C. acuminata* (**Figure 1**), it is CR that is likely to reflect the influence of DBH on leaf shape. It is apparent that more sun leaves grow in the upper canopy, and more shade leaves growth in the lower canopy. Sun leaves are smaller and thicker, and shade leaves are larger and thinner, and therefore mean leaf size increases from top to bottom (de Casas et al., 2011; Lian et al., 2023). However, leaf shape exhibits little variation along the longitudinal direction (Küppers, 1989). Thus, we argue that lower canopy samplings can reflect the morphological characteristics of leaves.

### 4.2 What has determined the numerical value of MP?

Shi et al. (2019) found that MP is related to leaf shape. Here, leaf shape means the planar geometry of a leaf. A triangular leaf has MP = 1/2, an elliptical leaf has MP = π/4, and a superelliptical leaf has (Gielis, 2003; Li et al., 2022a)


$$\text{MP = }\frac{4^{-1/n}\sqrt{\text{π}}\Gamma \left(1+1/n\right)}{\Gamma \left(0.5+1/n\right)}$$

where *n* is the parameter of the superellipse equation (Li et al., 2022a). Shi et al. (2021a) studied 10045 leaves from 101 bamboo taxa, and validated ME for the pooled data. The estimated value of MP for the 10045 bamboo leaves was equal to 0.696, which is smaller than π/4 and therefore falling between the triangular and the elliptical leaf shapes. Many Magnoliaceae species have leaf shapes that tend to be hypoellipses, i.e., superellipses where *n* < 2. Li et al. (2022b) studied 2220 leaves from nine species of Magnoliaceae, and found that the MP values ranged between 0.641 and 0.728. Schrader et al. (2021) and Ma et al. (2022) found that the MP values for some elliptical leaves were greater than π/4, which means that these leaf shapes tend to be hyperellipses, i.e., superellipses where *n* > 2. The present study shows that the MP value of ovate leaves for the pooled data of the six trees of *C. acuminata* equals 0.6419 (**Figure 5A**), which falls into the range (1/2, π/4), the previously reported empirical MP range of ovate leaves (Shi et al., 2019; Schrader et al., 2021). In summary, leaf shape determines the numerical value of MP. Although CR varied among the six trees, the MP value didn’t substantially change. Thus, DBH is not significantly correlated with MP. In addition, there is a need to note that the results could be significantly affected by the sample size. Moreover, the interaction between genetic and environmental factors may influence leaf shape expression. Finally, microclimatic conditions for the studied trees and leaves were not measured and analyzed in the present study, which are likely to have significant influences on leaf functional traits. It merits further investigation in future studies.

## 5 Conclusions

The present study examined the influence of diameter at breast height (DBH) on the leaf shape and leaf area of *C. acuminata* with typical ovate leaves. Three leaf-shape indices, including the leaf centroid ratio, the ratio of leaf width to length, and the leaf roundness index, were calculated for six trees with different DBH values. Based on the current experimental design and sample sizes, it was found that DBH only slightly influenced mean leaf centroid ratio, and did not increase or decrease the other two leaf-shape indices. The Montgomery equation (ME) was validated for the leaves of each tree, and DBH did not correlate with ME’s proportionality coefficient MP. The estimated value of MP for the pooled data was 0.6466, falling into the previously reported MP range of 1/2 to π/4. In addition, a power-law equation assuming that leaf area allometrically scales with the product of leaf length and width did not significantly enhance the goodness of fit compared with ME, the percentage error between ME and the power-law equation being smaller than 5%, obviating the need for a complex allometric relationship. The present study also suggests that there is no need to consider the influence of DBH on the proportional relationship between leaf area and the product of leaf length and width, which enables the investigator to sample leaves growing on trees easy to access.

## Data Availability

The original contributions presented in the study are included in the article/**Supplementary Material.** Further inquiries can be directed to the corresponding author.
